# Primary stability in total hip replacement: A biomechanical investigation

**DOI:** 10.1097/MD.0000000000008278

**Published:** 2017-10-20

**Authors:** Paul Schmitz, Boyko Gueorguiev, Ivan Zderic, Christian Pfeifer, Michael Nerlich, Stephan Grechenig

**Affiliations:** a Clinic of Trauma Surgery, University of Regensburg, Regensburg, Germany; b AO Research Institute Davos, Davos, Switzerland.

**Keywords:** locking screw hip stem, primary stability, screw fixation, total hip replacement

## Abstract

**Background:**

In total hip replacement (THR), it is essential to achieve a primary stability to guarantee good long-term results. A novel locking screw hip (LSH)-stem, anchored to the medial cortex of the proximal femur by 5 monocortical locking screws, was developed to overcome the shortcomings of uncemented press-fit and cemented straight stems while simultaneously achieving primary stability. The aim of this study was to investigate the biomechanical competence of the LSH-stem in comparison to an uncemented press-fit stem.

**Methods:**

Six pairs of embalmed human cadaveric femora from donors aged 68 to 84 years were assigned to 2 study groups (n = 6) with equal number of right and left bones. The specimens in each group and pair were implanted with either an uncemented press-fit stem or an LSH-stem and tested biomechanically under progressively increasing cyclic axial loading until catastrophic failure. Axial construct stiffness, failure load, and cycles to failure were detected and statistically evaluated at a level of significance *P* = .05.

**Results:**

Although the axial stiffness was comparable for both prosthesis types, the uncemented press-fit stem showed a significant lower stability in terms of failure load and cycles to failure in comparison to the LSH-stem, *P* = .04.

**Conclusion:**

Converting our results to percentage of bodyweight (BW) in an assumed adult patient of 80 kg shows that the LSH-stem achieves a primary stability allowing to carry average loads of up to 507% BW, whereas the uncemented press-fit stem carried average loads of up to 404% BW. We conclude that both stems achieve a primary stability strong enough to carry hip joint loads experienced in the immediate rehabilitation period after THR.

## 1 Introduction

Total hip replacement (THR) became a standard procedure not only for the treatment of coxarthrosis but also to treat advanced femoral head necrosis and femoral neck fractures. During the last 5 decades the number of THRs per year has steadily increased. Although THR is recognized as the most successful procedure of the 20th century in orthopedic and trauma surgery, it still bears the risk of failure.^[1]^ Development of uncemented hip stems commenced in the 1980s to address shortcomings of the cemented technique.^[2,3]^ Initial stability and initial lack of motion at the bone-prosthesis interface are essential for the achievement of good long-term results in primary hip replacement.^[4]^ Therefore, accuracy of host bone preparation and prosthesis design are crucial, whereas implant surface texture and quality of the bone-implant contact determine secondary stabilization.^[5–7]^

Even though locking screw-plate systems have revealed clear advantages in fracture fixation, locking screws have not been considered for the fixation of hip prosthesis stems so far.^[8]^ Recently, a “locking screw hip” (LSH)-stem (Scyon Orthopaedics AG, Au-Waedenswil, Switzerland) was developed as an alternative to conventional uncemented press-fit and cemented straight stems to achieve an immediate stability after its implantation.^[9]^

The aim of this study was to investigate the biomechanical behavior of the LSH-stem in comparison to an uncemented stem that claims to enhance primary stability in THR.^[10,11]^

## 2 Materials and methods

### 2.1 Specimens preparation and study groups

Six pairs human cadaveric femora (12 specimens in total, all males, mean age 74, range 68–84), embalmed with the method of Thiel,^[12]^ were used in the current study. All donors gave their informed consent within the donation of anatomical gift statement during their lifetime. None of the femora showed signs of previous injuries, abnormalities, or diseases.

All specimens were stripped of all soft tissue. Bone mineral density (BMD) of each specimen was evaluated in the femoral head by high resolution peripheral quantitative computed tomography using xTreme CT (Scanco Medical, Brütisellen, Switzerland). Scanning was performed at a resolution of 0.082 mm. BMD was calculated as mean value of scanned cortical and cancellous bone content within the volume of interest between 2 slices, perpendicular to the femoral neck axis and located at a distance 5 mm proximally and 5 mm distally to the centre of the femoral head, measured along this axis.

The specimen pairs were split to 2 study groups with 6 femora each, with equal numbers of right and left specimens per group, for implantation with either an uncemented press-fit stem (Spotorno equivalent, Scyon Orthopaedics AG, Au-Waedenswil, group 1) or an LSH-stem (Scyon Orthopaedics AG, Au-Waedenswil, group 2).

### 2.2 Implants

**Figure 1:**
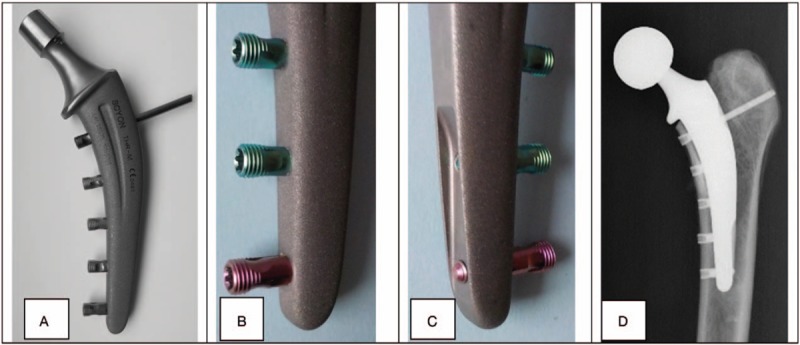
(A) Locking screw hip stem achieving primary stability by means of 5 mono-cortical locking screws attaching the stem to the proximal medial cortex of the femur. (B) Roughly grit-blasted medial side of the stem to allow good bone apposition onto the stem necessary for definitive stability. (C) The smooth lateral side of the stem leaves the lateral cortex untouched to avoid coupling between the medial and lateral cortex and thus prevent stress shielding in the trochanteric region. (D) X-ray of the locking screw hip stem as implanted to a cadaver femur.

The LSH-stem is made of titanium alloy and has a roughly grit blasted medial side to allow good bone apposition whereas the lateral side is kept smooth to avoid coupling between the medial and lateral cortex (Fig. 1). Primary stability is achieved by means of 5 monocortical locking screws fixing the stem to the proximal medial cortex of the femur.

The uncemented press-fit stem is an equivalent to the Spotorno stem that was developed by Spotorno in 1983 and became one of the most successful endoprosthesis.^[13,14]^ This stem is made of titanium alloy and has a complex proximal geometry of a double wedge-shaped core in combination with anterior and posterior vertical flutes. The entire stem is with a rough grit-blasted surface with a mean roughness of 3.5 μm (Ra).^[15]^ The stem is straight and achieves a primary stability by contacting the femoral cortex in at least 3 different areas.

### 2.3 Preoperative planning of the implantation

Digital X-rays were made for preoperative planning to simulate the technique for stem implantation as prescribed for clinical use. The planning for the uncemented press-fit stem implantation was performed with the use of MediCAD software (mediCAD HECTEC GmbH, Altdorf, Germany). Since currently there is no existing tool for planning of the LSH-stem implantation, femur size, intramedullary space, and resection line of the femoral neck were detected and measured with the same software for this prosthesis too. Specifically, all femora used for LSH-stem implantation were big enough so that the lateral edge of the stems did not come into contact with the lateral cortex of the femur, as desired by the manufacturer.

### 2.4 Prostheses implantation

All implantation procedures were performed by a single surgeon (PS). The femur shafts for the uncemented press-fit stem were prepared according to the current standards for endoprosthetic surgery. The femur shafts for the LSH-stem were prepared according to the surgical technique as prescribed by the manufacturer. Preoperative templates and surgical techniques ensured a proper alignment of the central axis of the stem with the long axis of the femur during implantation.

One LSH-stem size was required for the specimens in group 2. The stem size of the uncemented press-fit prosthesis (group 1) was calculated according to the preoperative planning and adjusted to the size of the reamer that achieved a proper grip in the femur during preparation. Intraoperative complications and any obvious femoral cracks were avoided by thorough preparation and careful implantation.

### 2.5 Postoperative analysis and specimens embedding for biomechanical testing

All specimens were analyzed postoperatively by computed tomography scanning in order to exclude any femoral damage. No fracture or fissures were detected. X-rays of the implanted prostheses were shot in order to measure the off-set and check the lever arm for each femoral pair using the software tool MediCAD. Center of rotation on the X-rays was detected with the help of Cobalt Chrome prosthesis heads with diameter of 32 mm, offset M (0.0 mm), and a 12/14 conus, which were attached to the prostheses prior to the X-ray shots. Following, the lengths of the femora were measured from the tip of the greater trochanter to the epicondyle line and the distal 3rd was resected. Then the distal end of each specimen was embedded in poly(methyl methacrylate) in preparation for biomechanical testing.

### 2.6 Biomechanical testing

**Figure 2:**
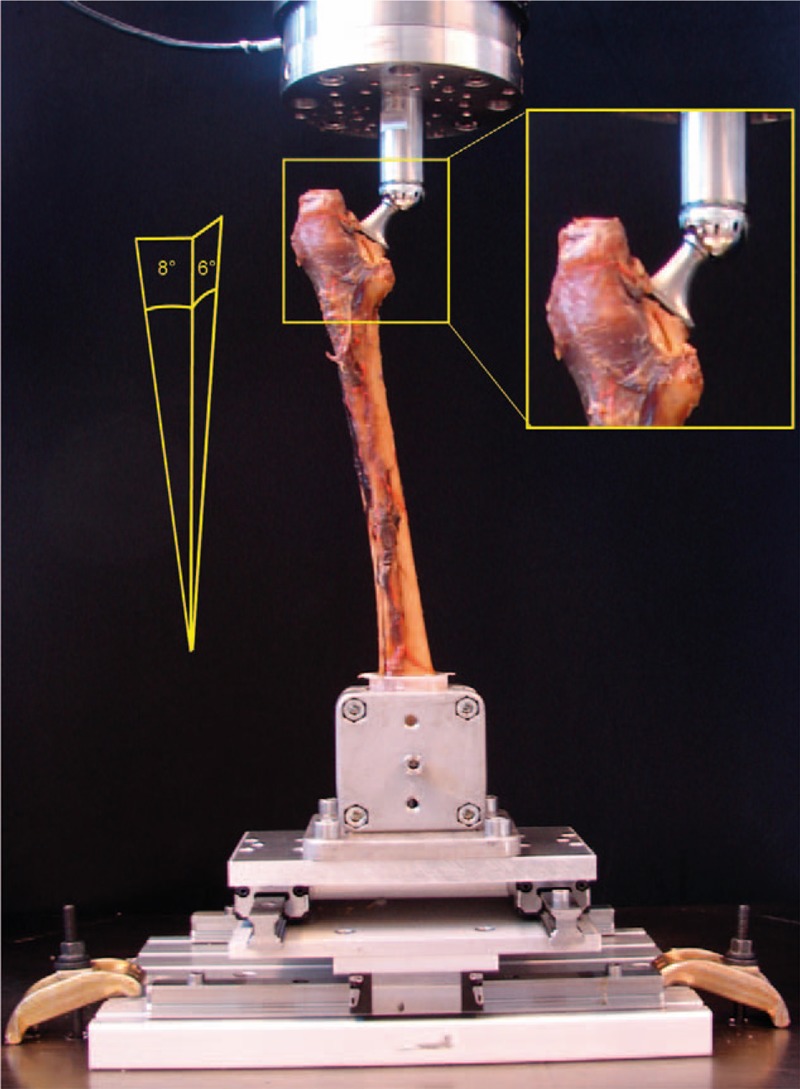
Setup with a specimen mounted for biomechanical testing.

Biomechanical testing was performed on a servohydraulic test system (Bionix 858.20; MTS Systems, Eden Prairie, MN) with a 25 kN/200 Nm load cell. The specimens were fixed in a specially designed rig for loading in cranio-caudal direction and tilted 8° lateral in the frontal plane and 6° dorsal in the sagittal plane to simulate a single leg stance loading^[11,16]^ (Fig. 2).

**Figure 3:**
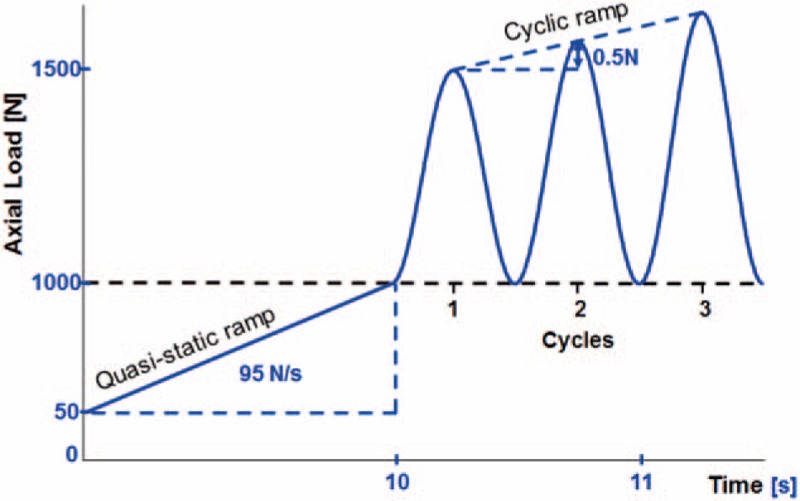
Mechanical testing protocol starting with a quasistatic ramped loading, beginning at 50 N preload until 1000 N in axial compression, followed by a cyclic ramp loading increasing by 0.5 N per cycle until catastrophic failure of the implant-bone-construct.^[17]^

Proximally, each specimen was attached to the machine actuator via a ball-and-socket joint. The embedded distal specimen's end was connected to the machine base via an XY table (Fig. 2). The testing protocol comprised a quasistatic and a cyclic loading part (Fig. 3). Quasistatic ramped loading to 1000 N was performed in axial compression at a rate of 95 N/s, starting from 50 N preload.^[17]^ The cyclic biomechanical test started from the final quasistatic loading condition and was performed at a rate of 2 Hz with sinusoidal axial loading. The 1st cycle ranged from 1000 N (valley) to 1500 N (peak). The peak level was then progressively increased by 0.5 N/cycle until construct failure occurred, as described in previous studies.^[17,18]^

### 2.7 Data acquisition and analysis

Machine data in terms of applied load and actuator displacement were recorded by the system transducers at a rate of 128 Hz. Anteroposterior X-rays were taken at the beginning of the test, then periodically at intervals of 500 cycles and after specimen's failure.

Based on the load-displacement curves, axial construct stiffness, failure load, and cycles to failure were defined as parameters of interest for each specimen and considered for statistical evaluation. Axial stiffness was calculated from the gradient of the respective load-displacement curve during the ramped loading in the quasilinear elastic region before plastic construct deformation occurred. Failure load, cycles to failure, and type of failure were defined from the destructive cyclic test.

### 2.8 Statistical analysis

Statistical analysis was performed using SPSS Statistics (Version 23, IBM SPSS, Armonk, NY). Normal distribution of the parameters of interest, namely BMD, axial construct stiffness, failure load, and cycles to failure in each study group was screened with Shapiro-Wilk test. Homogeneity of variances between the groups were checked with Levene test. Significant differences between the 2 groups were checked with paired-samples *t* test. Level of significance was set to *P* = .05 for all statistical tests.

## 3 Results

All parameters of interest (BMD, axial construct stiffness, failure load, and cycles to failure) were normally distributed in each of the study groups and with homogeneity of variances among the groups, *P* ≥ .37.

BMD (uncemented press-fit stem, group 1: 182.1 ± 53.9 mg HA/cm^3^ [mean ± standard deviation] and LSH-stem, group 2: 161.4 ± 33.6 mg HA/cm^3^) showed no statistical significant difference, *P* = .20.

Similarly, axial construct stiffness (uncemented press-fit stem, group 1: 1604.7 ± 376.1 N/mm and LSH-stem, group 2: 1953.6 ± 305.0 N/mm) did not differ significantly between the 2 groups, *P* = .10.

**Figure 4:**
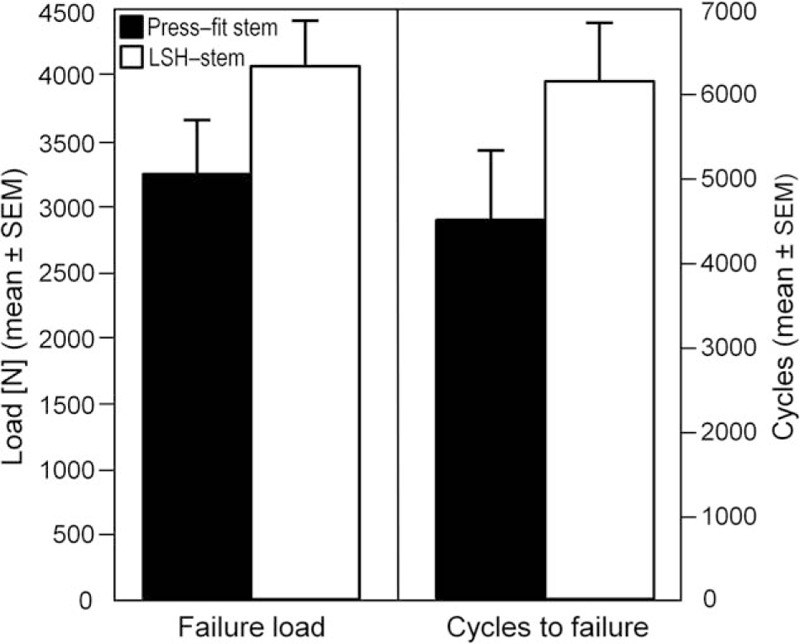
Mean values with SEMs of failure load and cycles to failure. LSH = locking screw hip, SEM = standard error of mean.

A statistically significant difference was detected in terms of failure load and cycles to failure between the uncemented press-fit stem, group 1 (failure load: 3232.2 ± 1009.2 N; cycles: 4464 ± 2018) and the LSH-stem, group 2 (failure load: 4061.1 ± 846.8 N, cycles: 6122 ± 1694), *P* = .04 (Fig. 4).

**Figure 5:**
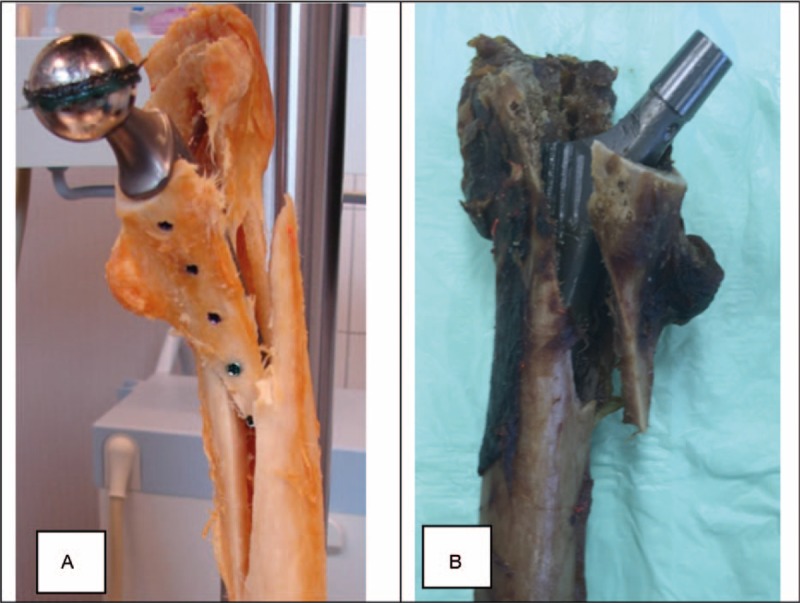
Fracture pattern of the proximal femur after catastrophic failure. (A) Locking screw hip (LSH)-stem: notice that even though the femur is broken the fixation of the prosthesis to the bone by the screws stays intact. (B) Uncemented press-fit stem.

A similar fracture pattern at catastrophic failure of the bone-implant construct was detected in most of the specimens (Fig. 5). A longitudinal crack at the ventral and/or dorsal proximal femur occurred predominantly in combination with an oblique fracture of the femoral diaphysis at the tip of the prosthesis. This led to bone split in the greater and minor trochanteric region as well as to separation between the proximal femur and the femoral diaphysis. An initial subsidence of the uncemented press-fit stem was detected visually for all specimens in group 1. Unexpectedly, no fracture occurred at the line of the screws fixing the LSH-stem to the femur, thus leaving the bone-implant construct intact for all specimens in group 2 (Fig. 5).

## 4 Discussion

In the current study, the normal distribution of the parameters of interest within the treatment groups supports the fact that the study sample can be representative for a population which has a normal distribution of those data. Moreover, the application of parametric statistical test for the detection of significant differences between the groups, which is justified by this normal distribution, aimed to achieve higher statistical power during the data analysis.

Even though a numerous amount of publications emphasize the good long-term outcomes of THR, endoprosthetic register data shows a high number of hip stems that need revision surgery due to aseptic loosening. One reason for the aseptic loosening is subsequent stress shielding in the trochanteric region occurring years after THR. The reason for stress shielding is not yet completely understood; however, the following facts seem to be in place for it. First, the lateral cortex and lateral cancellous bone of the proximal femur, as well as the gluteal muscles attached at the greater trochanter, are compromised during implantation, especially when lateral or anterolateral approach to the hip joint are used. Second, through the design of the common hip stems, a coupling between the medial and lateral cortex is created, leading to a rather nonphysiological transfer of the loads at the hip joint.

Another reason for aseptic loosening of a hip stem is continual micromotion occurring prior to settling of solid prosthesis anchorage via bone in/ongrowth.^[4]^ Achieving a primary stability of the hip stem can avoid the formation of a fibrous layer at the bone-prosthesis interface and enhance bone in/ongrowth to the prosthesis.^[19]^

To overcome these issues, a new hip stem design – the LSH-stem – was developed by Scyon Orthopedics AG. The concept of the LSH-stem targets achievement of primary stability by using 5 monocortical locking screws transferring joint forces directly to the femur through the medial calcar and reproducing a more physiological load situation. In addition, a coupling between the medial and lateral cortex can be avoided by the size of the stem since it does not have to fill the medullary cavity to achieve primary stability.

Even though the concept of the LSH-stem seems very promising, it is still not known how well the use of 5 monocortical locking screws, fixing the stem to the medial cortex of the proximal femur, will withstand the hip joint forces that occur until bone remodeling provides a solid prosthesis support.

This unknown was the motivation to perform ex vivo experimental investigations to analyze the biomechanical behavior and to compare the biomechanical performance of the LSH-stem to another uncemented stem for THR.

By using a human cadaveric model in a combined matched paired design, the influence of anatomical variability in the specimen's parameters such as femur size, curvature, and cortex thickness was considerably neutralized. Thus, a much better comparison of the intrinsic biomechanical performance of the 2 prosthesis types was possible. The 2 following facts became obvious.

First, no statistically significant difference between the novel LSH-stem and the uncemented press-fit stem was found in terms of axial construct stiffness under single-leg stance loading conditions. Second, the failure load and cycles until failure for the LSH-stem were significantly higher than the uncemented press-fit stem. In a previous study with the same setup for biomechanical testing, Grechenig et al^[9]^ reported comparable axial construct stiffness and failure load for the LSH-stem and a cemented straight stem. This means that the biomechanical properties of the novel LSH-stem are comparable to the cemented straight stem in terms of primary stability, and even superior to the uncemented press-fit stem.

An uncemented hip stem reaches its maximum stability after bone ingrowth.^[20]^ Besides the appropriate surface structure, application of bioceramic coatings can contribute to a good mechanical fixation of an endoprosthetic implant.^[6]^ The LSH-stem is designed with a rough grit-blasted medial side to allow good bone apposition and integration for definitive stability. In a radiostereometric analysis study including 15 patients with implanted LSH-stems, aged 50 years on average, the successful transformation of primary stability to secondary stability could be shown.^[21]^ Taking into account these results and the fact that according to the findings of Bergmann et al^[1,19]^ the experimental setup we chose seems to be a quite realistic or even processing the worst case scenario to test hip implants with regard to their stability of fixation during the immediate postoperative phase, the clinical application of the LSH-stem may be justified.

### 4.1 Limitations

The limitations of this study are similar to those inherent to most cadaveric biomechanical studies with a limited number of tested specimens, small sample size, and scattering of the measured data, thus rendering the statistical power. In addition, the use of embalmed specimens in comparison to fresh-frozen specimens might have influenced the results since Unger et al^[22]^ detected a reduction of the Young modulus for embalmed cadaver bone.
